# Antarctic killer whales make rapid, round-trip movements to subtropical waters: evidence for physiological maintenance migrations?

**DOI:** 10.1098/rsbl.2011.0875

**Published:** 2011-10-26

**Authors:** J. W. Durban, R. L. Pitman

**Affiliations:** Protected Resources Division, Southwest Fisheries Science Center, National Marine Fisheries Service, National Oceanic and Atmospheric Administration, 3333 North Torrey Pines Court, La Jolla, CA 92037, USA

**Keywords:** migration, killer whales, Antarctica

## Abstract

Killer whales (*Orcinus orca*) are important predators in high latitudes, where their ecological impact is mediated through their movements. We used satellite telemetry to provide the first evidence of migration for killer whales, characterized by fast (more than 12 km h^−1^, 6.5 knots) and direct movements away from Antarctic waters by six of 12 type B killer whales tagged when foraging near the Antarctic Peninsula, including all tags transmitting for more than three weeks. Tags on five of these whales revealed consistent movements to subtropical waters (30–37° S) off Uruguay and Brazil, in surface water temperatures ranging from −1.9°C to 24.2°C; one 109 day track documented a non-stop round trip of almost 9400 km (5075 nmi) in just 42 days. Although whales travelled slower in the warmest waters, there was no obvious interruption in swim speed or direction to indicate calving or prolonged feeding. Furthermore, these movements were aseasonal, initiating over 80 days between February and April; one whale returned to within 40 km of the tagging site at the onset of the austral winter in June. We suggest that these movements may represent periodic maintenance migrations, with warmer waters allowing skin regeneration without the high cost of heat loss: a physiological constraint that may also affect other whales.

## 1. Introduction

Killer whales (*Orcinus orca*) are important marine predators, especially in high latitudes, where energetic calculations suggest that their high caloric requirements could significantly impact slowly reproducing prey populations [1]. In Antarctic waters, where killer whales are particularly abundant [2], predation by them may be a major force structuring marine communities [3,4]. However, their ecological impact must be mediated by their movement and residency patterns, which are largely unknown in this region [5].

Although killer whales are known to range over thousands of kilometres [6,7], to date there is little direct evidence of seasonal or regular long-distance migrations anywhere, including Antarctica [8]. Here, we present movement data from type B killer whales, predators of ice seals and penguins [9,10], which were satellite-tagged in Antarctica. We document an unprecedented, rapid migration to subtropical latitudes and back. The evidence suggests that these movements were not for breeding or feeding purposes, and we hypothesize that they might be physiologically adaptive.

## 2. Material and methods

Satellite tags were deployed on type B [8] killer whales in two areas off the west coast of the Antarctic Peninsula, during January 2009–February 2011: in Laubeuf Fjord, east of Adelaide Island, on whales that were feeding primarily on Weddell seals (*Leptonychotes weddellii* [10]); and in the Gerlache Strait, east of Anvers Island, where they were observed to feed on pygoscelid penguins [9]. Visual searches were conducted from a motor-sailing yacht and expedition ship, and close approaches were made from a 4.0 m Zodiac launch to deploy ‘dart’ tags [5] with satellite transmitters (SPOT5 model, Wildlife Computers, Redmond, WA, USA; http://www.wildlifecomputers.com/). These small (49 g) tags were attached with two barbed titanium posts which penetrated 6.5 cm into the dorsal fin. Tags were deployed using a crossbow bolt fired at ranges from 5 to 15 m using a crossbow of 150 lb draw weight; the bolt fell away on contact with the whale, leaving only the tag attached. Tags were scheduled to transmit up to 600 times during four 3 h periods each day, and locations were calculated by the ARGOS satellite system using the method of least squares (CLS America, Largo, MD, USA; http://www.argos-system.org/).

Outliers from the estimated tag locations were removed using a filtering algorithm [11] with a maximum swim speed of 25 km h^−1^, and a continuous time correlated random walk model [12] was fitted to the remaining locations to estimate displacement velocities. Temporal trends in velocities for each tag were estimated by fitting a Bayesian regression model with unknown change-points ([13]; electronic supplementary material, S1), and these were then related to latitudinal changes over time. Track locations were spatially linked to sea surface temperature (SST) using the Intersect Point Tool of the Hawth's Tools extension (http://www.spatialecology.com/) for software ESRI ArcMap v. 9.3 (http://www.esri.com/), using a blended SST dataset derived from microwave and infrared sensors carried on multiple spacecraft platforms (http://coastwatch.pfel.noaa.gov/infog/BA_ssta_las.html/). SST data were available at a spatial resolution of approximately 11 km, and we used a 5 day window around the date of the lowest latitude location for each whale.

## 3. Results

Tags were deployed on 12 type B killer whales, including 10 adult females, one subadult male and one adult male (table 1), in groups of 4–70 (median = 14). A total of 11 261 locations were obtained over a combined 553 transmission days (mean duration = 46 days, range = 9–109). Two whales tagged in the same group (whales 6 and 7) remained together for a minimum of 98 days.

**Table 1.** Details of type B killer whales tracked with satellite tags, including the minimum latitude and maximum sea surface temperature (SST) recorded along the track.

whale	age/sex	year	tag duration (days)	min. latitude (° S)	max. SST (°C)
1	adult/female	2009	08 Jan–16 Jan (9)	64	2.3
2	adult/female	2009	14 Jan–27 Jan (14)	67	2.1
3	adult/female	2009	15 Jan–31 Jan (17)	60	2.9
4	adult/female	2009	24 Jan–13 Feb (21)	51	11.2
5	subadult/male	2010	13 Feb–24 Feb (12)	63	2.1
6	adult/female	2010	13 Feb–21 May (98)	30	22.4
7	adult/female	2010	13 Feb–01 Jun (109)	30	22.4
8	adult/male	2011	13 Jan–31 Jan (19)	67	1.8
9	adult/female	2011	13 Jan–25 Feb (44)	37	22.9
10	adult/female	2011	15 Jan–16 Apr (93)	36	20.9
11	adult/female	2011	15 Jan–22 Apr (99)	30	24.2
12	adult/female	2011	24 Jan–13 Feb (21)	65	1.9

**Figure 1.**
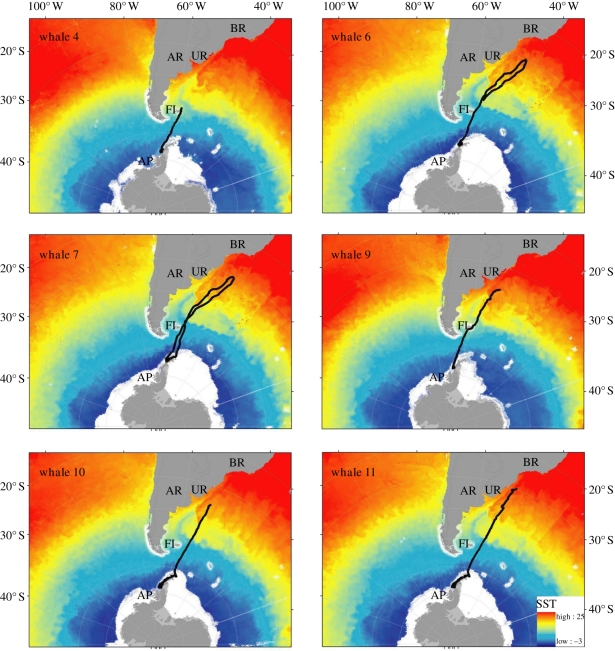
Tracks (black line) from satellite tags on six type B killer whales (table 1) that ranged away from the Antarctic Peninsula (AP), displaying consistent movements past the Falkland Islands (FI) and Argentina (AR), to and from lower latitudes off Uruguay (UR) and Brazil (BR), with warmer sea surface temperatures (SST, °C).

**Figure 2.**
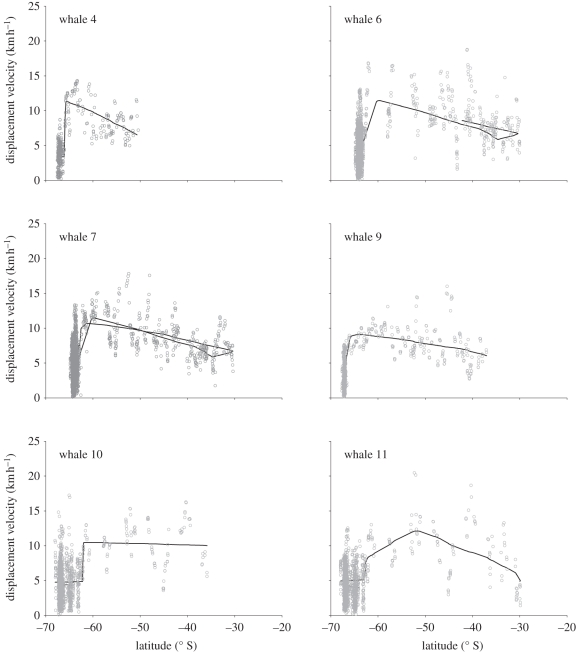
Variation in displacement velocities (open circles) by latitude for six satellite-tagged type B killer whales (table 1) that moved out of Antarctic waters (less than 60° S): solid line represents the average velocity described by a Bayesian piecewise regression model of velocity against time (electronic supplementary material, S1).

In coastal waters of the Antarctic Peninsula where the whales were observed hunting [9,10], movements were characterized by frequent changes in direction and relatively low net displacement (figure 2); velocities averaged between 3 and 4.7 km h^−1^ and SST ranged from −1.9°C to 2.9°C. Six of the 12 tags, comprising all that lasted more than three weeks, then indicated a marked change in whale behaviour with an abrupt shift to rapid northward travel out of Antarctica (less than 60° S) into open ocean waters (figure 1). The Bayesian regression model clearly identified a change in swim speed, with average velocities increasing to more than 9 km h^−1^ in all cases (range = 9.12–12.13; figure 2). These departures took place over an 80 day period between 4 February and 20 April over three different years (electronic supplementary material, S1).

These six whales all followed a consistent route into the southwest Atlantic, passing just east of the Falkland Islands (figure 1). Tags on 5/6 of these whales revealed non-stop movements to subtropical waters (30–37° S) off the coasts of Uruguay and southern Brazil, to SSTs ranging from 20.9°C to 24.2°C. Two of these whales (6 and 7) began travelling south before the tags ceased transmitting, and one (7) completed a 42 day round trip of 9392 km, before returning to Antarctica on 1 June (SST −1.9°C) to a location 40 km from where it was tagged. A second group that left in February 2009 (4) was subsequently photographed back around the Antarctic Peninsula in June 2010 (A. Friedlaender 2010, unpublished data) and again in January 2011 by the authors.

After leaving Antarctic waters at relatively high speed, all six longest-lasting tags showed declining velocity with decreasing latitude, to average speed of 5–10 km h^−1^ at varying minimum latitudes (figure 2). Notably, the two returning whales exhibited a corresponding increase in velocity as they followed a very similar track back to Antarctica, with the whale 7 reaching average speed in excess of 10 km h^−1^ south of 52°, as it crossed the Drake Passage between the Falkland Islands and the Antarctic Peninsula. Once back in the coastal waters near the tagging site, it abruptly slowed down to ‘foraging speed’ less than 5 km h^−1^ on the last transmission day (mean = 4.2, range 3.5–4.7).

## 4. Discussion

Despite frequent sightings in Antarctic waters, there have been very few records of type B killer whales occurring elsewhere [8]. Our results, however, suggest that they regularly travel to the subtropics, thus providing the first documentation of directed, long distance migration for killer whales anywhere. Although these migrations will limit pressure on prey around Antarctica, they could lead to short term increases in local predation if whales are ‘topping off’ before they depart [10].

This migration was characterized by a consistent and directed progression to and from lower latitudes at displacement speed higher than when foraging around the Antarctic Peninsula. Remarkably, one whale returned to Antarctica after completing a 9400 km trip in just 42 days. We suggest that the speed and duration of this trip would have left little time for prolonged foraging, and probably also would have been too challenging for a newborn calf. Most large scale whale migrations are currently explained as movements for feeding or breeding purposes [14], but, as an alternative, we suggest that this migration we documented may represent a physiological maintenance migration. Our tagged whales followed the most direct path to the nearest warm waters north of the subtropical convergence, with a gradual slowing of swim speed in progressively warmer water. We therefore hypothesize that these migrations were thermally motivated, and they might be facultative rather than seasonal because they were initiated across a range of at least 80 days between February and April, with at least one whale returning to cold waters (−1.9°C) at the onset of the austral winter in June.

Killer whales are the smallest cetaceans that regularly occur in subzero Antarctic waters, and this extreme thermal environment presents them with significant physiological challenges [15], including repairing and replacing their outer skin layer while maintaining thermal integrity. Similar thermal considerations underlie the requirement for pinnipeds to haul-out on land to moult, and the annual movement of beluga whales (*Delphinapterus leucus*) into warmer water estuaries in the Arctic [16]. As evidence for limited skin regeneration, killer whales in Antarctica are often coloured yellow because of a thick accumulation of diatoms on their skin [8], but the same animals are at other times ‘clean’ (electronic supplementary material, figure S2). We infer that the latter have recently returned from warmer waters where they shed their diatoms while replacing their epidermal tissue. If this hypothesis should prove true, it could have implications for migratory movements of other killer whale ecotypes in high latitudes [7], and perhaps other large whales as well. Currently, there is no consensus on why baleen whales migrate to the tropics [14], but we suggest that similar physiological constraints could be a factor.
